# The Effect of Intraoperative Dexmedetomidine on Postoperative Delirium Sedation Agitation Score in cardiac surgery

**DOI:** 10.5812/aapm-156544

**Published:** 2025-03-11

**Authors:** Mohammad Hosein Ghanbarpour, Ali Dabbagh, Alireza Jahangirifard, Navid Shafigh, Mina Fani, Kamal Fani

**Affiliations:** 1 Department of Anesthesiology, School of Medicine, Zanjan University of Medical Sciences, Zanjan, Iran; 2 Department of Anesthesiology, Anesthesiology Research Center, School of Medicine, Shahid Beheshti University of Medical Sciences, Tehran, Iran; 3 Lung Transplantation Research Center, National Research Institute of Tuberculosis and Lung Diseases (NRITLD), Shahid Beheshti University of Medical Sciences, Tehran, Iran; 4 Department of Anesthesiology and Critical Care Medicine, School of Medicine, Shahid Beheshti University of Medical Sciences, Tehran, Iran; 5 School of Medicine, Shahid Beheshti University of Medical Sciences, Tehran, Iran; 6 Department of Anesthesiology, School of Medicine, Shahid Beheshti University of Medical Sciences, Tehran, Iran

**Keywords:** Dexmedetomidine, Postoperative Delirium, Cardiac Surgery

## Abstract

**Background:**

Postoperative delirium is a significant problem that deteriorates the cognitive state of patients after cardiac surgery, which can be a short- or long-term complication.

**Objectives:**

This study was conducted to evaluate the efficacy of dexmedetomidine, commenced simultaneously with anesthesia induction and continued throughout the surgical operation, on postoperative delirium after cardiac surgery with cardiopulmonary bypass.

**Methods:**

This randomized, double-blind, case-control trial was conducted on sixty-one patients undergoing cardiac surgery. The patients were randomly divided into dexmedetomidine (case) and normal saline (control) groups. The primary outcome was the incidence of delirium, as screened by the Confusion Assessment Method for the ICU (CAM-ICU).

**Results:**

There was no distinction in CAM-ICU outcomes between the two groups at 6 and 24 hours postoperatively. However, the difference in non-positive CAM-ICU results was statistically significant at 24 hours for +1 and -1 Richmond Agitation-Sedation Scale scores.

**Conclusions:**

Starting dexmedetomidine before cardiopulmonary bypass did not significantly affect the delirium rate based on CAM-ICU assessments. Further research examining larger groups is necessary to clarify the efficacy of perioperative dexmedetomidine on postoperative delirium.

## 1. Background

Postoperative delirium (POD) is a prevalent and debilitating complication after major surgery, with its occurrence after cardiac surgery reported to be as high as 52% (1). It is characterized by an acute decline in cognitive function, including inattention, altered consciousness, and disruption of normal activity (2). The pathophysiology of delirium is not entirely understood but likely involves neuroinflammation, oxidative stress, neurotransmitter imbalance, and medications (3). Patients undergoing cardiac surgery are more susceptible to POD due to the extensiveness of the operation, underlying diseases, and the specific age of the patients, especially in coronary artery bypass graft (CABG) surgery (4). Additionally, the use of cardiopulmonary bypass (CPB) likely increases the risk of POD. Delirium is associated with multiple adverse consequences, including prolonged intensive care unit (ICU) and hospital stays, increased healthcare costs, persistent cognitive decline, and greater mortality (5). However, no pharmacological agent has firmly demonstrated efficacy for delirium prevention and treatment so far (6).

Dexmedetomidine, a highly selective alpha-2 adrenoceptor agonist, is one of the most extensively studied drugs for managing delirium due to its sedative and analgesic properties without significant respiratory depression (7). Several randomized trials have evaluated intraoperative or postoperative dexmedetomidine for delirium prevention in cardiac surgery patients, but the results remain inconsistent and controversial. While some studies revealed decreased POD with dexmedetomidine (8), others showed no significant benefit compared to placebo (9, 10). These conflicting findings may be related to differences in study design, patient population, and dexmedetomidine dosing regimens. Given the clinical and economic burden associated with POD, it is essential to find safe and effective strategies to prevent this complication.

This randomized, double-blind, case-control study aimed to evaluate the effect of intraoperative dexmedetomidine infusion initiated after anesthesia induction on POD incidence in patients undergoing CABG surgery. We also assessed its impact on postoperative sedation and agitation using validated tools like the Richmond Agitation-Sedation Scale (RASS) and the Confusion Assessment Method for ICU (CAM-ICU). POD is often perceived as a brief attenuation of brain function with complete remission; however, it is, in reality, a severe problem that deteriorates the cognitive state of patients after cardiac surgery, which can be a short- or long-term complication and is occasionally associated with prolonged hospital stays (11). Agitation and sedation are difficult to define and manage. Agitation is a state of motor and cognitive hyperactivity marked by inappropriate or excessive verbal or motor activity, as well as emotional excitement (12).

Delirious patients might be confused or agitated and can appear alert or drowsy. According to the findings of Boettger et al., drowsiness increased the odds of developing delirium eightfold and caused more severe delirium, characterized by sleep-wake cycle and language abnormalities (13). On the other hand, agitation can manifest similarly to delirium or be a prelude or part of it. Isolated agitation is a benign complication, but delirium can be fatal (14). Agitation and confusion do not necessarily correspond to delirium, but the severity of deviation from the optimal state (calm and responsive) brings the patient increasingly closer to the definition of delirium.

Based on results from randomized trials, no indication has been found to support the role of any drug as a preventive factor for POD (15, 16), although dexmedetomidine may be a possible exception. Dexmedetomidine is a highly selective α2-adrenoceptor agonist that induces sedation with minimal respiratory depression and possesses analgesic-sparing, anxiolytic, and sympatholytic properties (17). Furthermore, due to its effect on minimizing ICU stay length and decreasing the need for mechanical ventilation, dexmedetomidine is suggested for sedation of mechanically ventilated patients in the ICU (18). Bradycardia and hypotension are common side effects of this sedative, but multiple studies have demonstrated that perioperative dexmedetomidine administration can reduce the incidence of delirium in patients undergoing cardiac surgery (19), although several other studies have not found supporting evidence (20, 21). There is no consensus on whether dexmedetomidine is useful for postoperative delirium (22). 

## 2. Objectives

This study was conducted to measure the efficacy of dexmedetomidine applied at induction of anesthesia and continued until the end of surgery on POD after cardiac surgery with CPB. We hypothesize that intraoperative dexmedetomidine infusion will reduce the incidence of postoperative delirium compared to normal saline.

## 3. Methods

Ethical approval was obtained with permission number IR.SBMU.RETECH.REC.1398.332 from the institutional review board. The study was registered in the Iranian Registry of Clinical Trials (IRCT20180724040575N4), and written informed consent was obtained from all participants. Data collection for this study was conducted in a tertiary teaching hospital. This randomized, double-blind, case-control trial involved 61 patients aged 40 - 70 years with an ejection fraction (EF) > 40% who were undergoing elective CABG surgery. Patients were randomly assigned to receive either intraoperative dexmedetomidine infusion (case group) or normal saline infusion (control group) using block randomization and a computer-generated random number table.

Anesthesia was induced with fentanyl, midazolam, propofol, and atracurium, and trial infusions started after anesthesia induction and continued until the end of surgery. The dexmedetomidine infusion was prepared as 200 μg dexmedetomidine (Exir^®^) in 50 mL normal saline to a final concentration of 4 μg/mL, and infused at a rate of 0.2 - 0.5 μg/kg/h. Based on FDA approval, the recommended dose for procedural maintenance infusion of dexmedetomidine is to initiate at 0.6 mcg/kg/hour and titrate with dosages ranging from 0.2 to 1 mcg/kg/hour (23). To avoid the side effects of dexmedetomidine in mostly elderly patients undergoing cardiac surgery, we limited the drug dose to the lower half of the range. The control group received normal saline at 0.1 mL/kg/h.

The primary outcome measure was postoperative delirium, assessed by the CAM-ICU at 6 and 24 hours after surgery. Secondary outcomes included RASS scores, hemodynamic parameters, ICU length of stay, and postoperative complications. All outcomes were assessed by blinded investigators.

### 3.1. Sample Size Calculation 

The sample size was calculated based on the primary outcome, postoperative delirium incidence, assessed using the CAM-ICU. A 30% difference in delirium incidence between the dexmedetomidine and control groups was considered clinically significant. Assuming a delirium incidence of 40% in the control group and a 10% lower incidence in the dexmedetomidine group, the required sample size was determined using the standard formula for comparing two proportions.


$$n=\frac{{\left(Z_{\frac{\alpha }{2}}+Z_{\beta }\right)}^{2}\times \left[P_{1}\left(1-P_{1}\right)+P_{2}\left(1-P_{2}\right)\right]}{({P_{1}-P_{2})}^{2}}$$

Where:

Z_α/2_ = 1.96 (for a 95% confidence level) Z_β _= 0.84 (for an 80% power); P_1_ = 0.40 (expected delirium incidence in the control group); P_2_ = 0.10 (expected delirium incidence in the dexmedetomidine group); P_1_ - P_2_ = expected difference (30%); Control group delirium incidence = 40%; Dexmedetomidine group expected incidence = 10% lower

Then substituting the values:


$$n=\frac{{\left(1.96+0.84\right)}^{2}\times \left[\left(0.4\times 0.6\right)+\left(0.1\times 0.9\right)\right]}{{\left(0.4-0.1\right)}^{2}}$$

$$n=\frac{{\left(2.8\right)}^{2}\times \left[\left(0.24\right)+\left(0.09\right)\right]}{{\left(0.3\right)}^{2}}$$

$$n=\frac{7.84\times 0.33}{0.09}$$

n = 28.8 (about 29) in each group

To account for potential dropouts, the final sample size was increased by 20%, leading to a target of 35 patients per group. Thus, a total of 70 patients were planned for enrollment in the study.

### 3.2. Inclusion and Exclusion Criteria

Patients were included if they had an ejection fraction (EF) above 40%. We excluded cases with known allergies to dexmedetomidine, those who took antipsychotic drugs, or had a history of delirium, neuropsychiatric illness, and seizures. Patients with an indication for emergency operation were also not included.

### 3.3. Randomization and Blinding

The study was conducted in a double-blind manner. Participants, clinical staff, and the intensivist who assessed the patients for the presence of delirium were blinded to the study groups. Only the physician responsible for the patient's anesthesia was aware of the study group to manage any potential hemodynamic complications. Block randomization and a computer-generated random number table were used for randomization into two groups.

### 3.4. Participants

A total of 61 adult patients aged 40 to 70, with American Society of Anesthesiologists (ASA) physical status II - III, who were scheduled to undergo CABG surgery were included. All patients were premedicated with lorazepam (1 mg) the night before surgery. Upon arrival in the operating room, standard monitoring, including pulse oximetry, invasive blood pressure monitoring, electrocardiogram, capnometry, and cerebral oximetry, was applied. Induction of anesthesia was performed using propofol (2 mg/kg), atracurium (0.5 mg/kg), fentanyl (0.7 μg/kg), and midazolam (0.1 mg/kg), followed by tracheal intubation. Anesthesia was maintained with isoflurane during the surgery. Participants were randomly divided into two groups (case and control) using a computer-generated random number table. The case group received dexmedetomidine infusion during cardiac surgery, while the control group received normal saline infusion as a placebo.

### 3.5. Cases

In the case group, after induction of anesthesia, dexmedetomidine was administered at a dose of 0.2 to 0.5 micrograms per kilogram per hour as an intravenous infusion and continued until the end of surgery. We used dexmedetomidine manufactured by Exir, with an initial dose of 200 micrograms in 2 mL, which was dissolved in 50 mL of normal saline produced by Shahid Ghazi Serum Manufacturing Company, resulting in a final concentration of 4 micrograms per mL.

### 3.6. Controls

In the control group, after induction of anesthesia, normal saline was administered at a dose of 0.1 mL/kg/h as an intravenous infusion until the end of the operation.

### 3.7. Outcome Measures and Statistical Analysis

After completion of surgery, patients were transferred to the ICU. Postoperative delirium and sedation-agitation conditions were measured using the CAM-ICU and the RASS, respectively. Patients were evaluated 6 and 24 hours after their operation for delirium, agitation, and sedation. Intubation time, length of ICU stay, postoperative ejection fraction (EF), hemodynamic variables, and total use of drugs such as inotropes, sedatives, narcotics, and sodium bicarbonate were documented as secondary outcomes.

### 3.8. Statistical Analysis 

Statistical analysis was performed using SPSS version 22 (IBM Corp., Armonk, NY, USA). Continuous variables were assessed for normality using the Shapiro-Wilk test. Normally distributed continuous data were expressed as mean ± standard deviation (SD) and compared between groups using the independent *t*-test. Non-normally distributed data were reported as median (interquartile range, IQR) and compared using the Mann-Whitney U test. Categorical variables were expressed as counts and percentages and analyzed using the Chi-square test (χ² test). If any expected frequency was less than 5, Fisher’s exact test was used.

For the primary outcome (incidence of postoperative delirium assessed by CAM-ICU), a Chi-square test (or Fisher’s exact test if required) was used to compare the proportion of patients with delirium between the dexmedetomidine and control groups. For the secondary outcome (sedation and agitation levels assessed by the RASS), the Mann-Whitney U test was used, as RASS scores are ordinal variables. Intraoperative and postoperative characteristics were compared between groups using the appropriate statistical tests (independent *t*-test, Mann-Whitney U test, or chi-square test) based on data type and distribution.

If applicable, correlation analysis between continuous variables (e.g., RASS score and ICU length of stay) was performed using Pearson correlation (for normally distributed variables) or Spearman correlation (for non-normally distributed variables). A multivariate logistic regression analysis was planned to adjust for potential confounders (e.g., age, EF, duration of surgery) in predicting postoperative delirium. The results were reported as odds ratios (OR) with 95% confidence intervals (CI). A P-value < 0.05 was considered statistically significant.

## 4. Results

**Figure 1.**
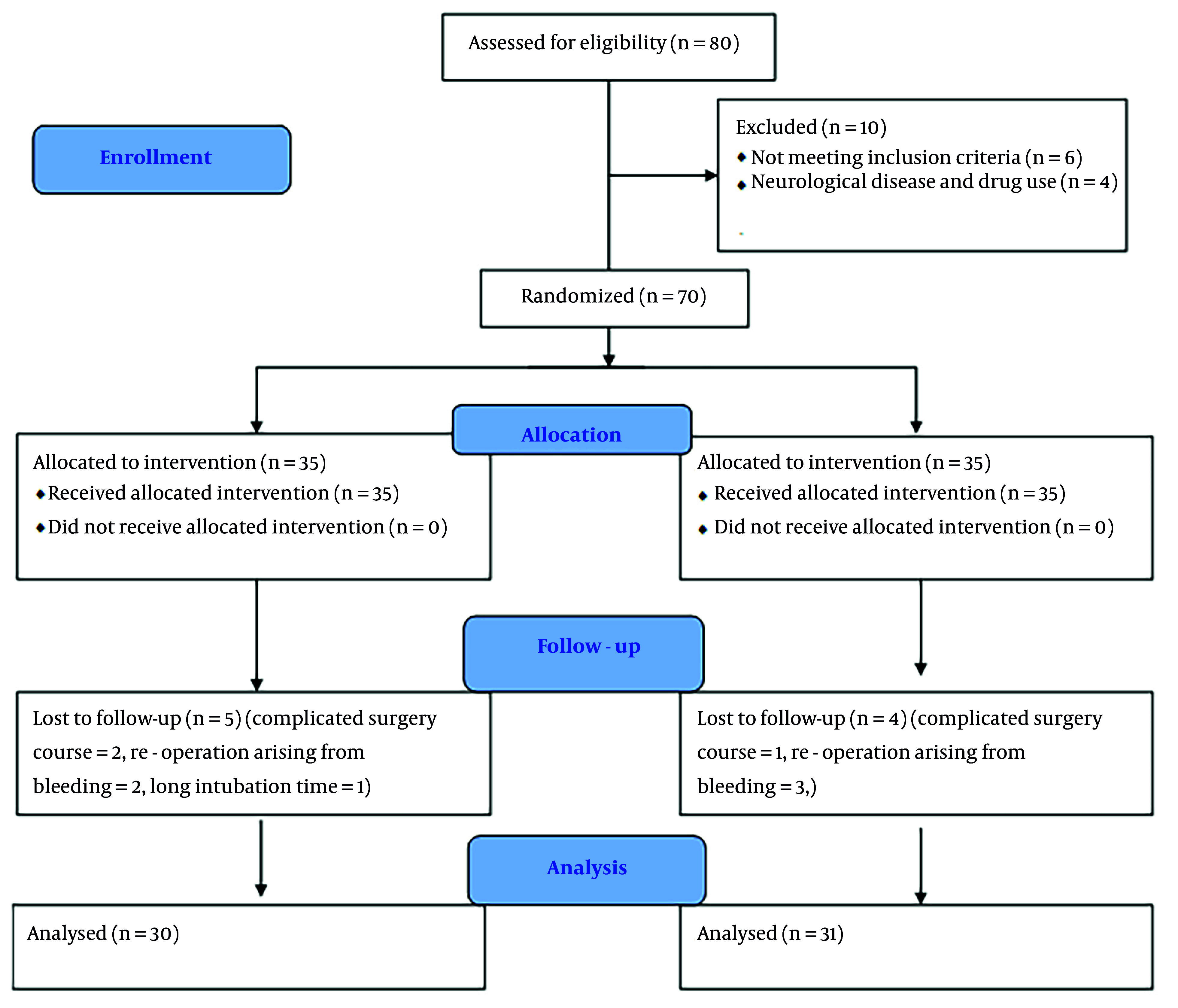
CONSORT flow diagram

A total of 61 patients, consisting of 15 women and 46 men, were the final subjects included in the present study. Initially, 70 patients were planned for inclusion. To reach this number, 80 patients were assessed for eligibility. Of these, 6 patients were not included due to not meeting the inclusion criteria, and 4 patients were excluded due to neurological diseases and drug use. Of the patients who entered the study, 9 were excluded during the study due to complicated surgery courses, re-operation arising from bleeding, and prolonged intubation time for various reasons. Data analysis was conducted on all 61 patients (Figure 1). The baseline characteristics of the two groups, including age, gender, weight, height, past medical history, and preoperative medications, were similar, with no significant differences between the dexmedetomidine and control groups (Table 1). The intraoperative characteristics, such as pump time, cross-clamp time, amount of narcotics, benzodiazepines, blood products transfusion, urine output, and inotrope doses, were also comparable between the two groups, with no significant differences observed (Table 2). The opioid equivalent table was used to record the amount of narcotics used based on morphine (24). Since the benzodiazepine used in all patients was exclusively midazolam, the total dose of benzodiazepine was recorded as equal to the dose of midazolam.

**Table 1.** Overall Characteristics of Included Cases and Controls

Characteristic	Case (n = 30)	Control (n = 31)	P-Value
**Age (y)**	63 ± 10	60 ± 7	0.2
**Weight (kg)**	75 ± 22	72 ± 11	0.5
**Height **	166.8 ± 8	168.5 ± 6.3	0.3
**Gender**			0.4
Male	24	22	
Female	6	9	
**Past medical history**			
Diabetes mellitus	8	12	0.3
Hypertension	13	19	0.1
Hyperlipidemia	10	15	0.2
Chronic kidney disease	1	2	0.5
EF (%)	48 ± 10	49 ± 9	0.6
PAP (mmHg)	30 ± 7	29 ± 6	0.4
Addiction	8	8	0.9
Smoking	8	13	0.2
**Drug history**			
Plavix	9	6	0.3
losartan	10	14	0.3
Aspirin	13	19	0.1
Statin	18	16	0.7
Beta-blocker	15	21	0.1
Ace-inhibitor	6	5	0.6

Abbreviations: EF, ejection fraction; PAP, pulmonary artery pressure.

**Table 2.** The Comparison of Intra-Operative Characteristics Between Cases and Controls

Characteristic	Case (n = 30)	Control (n = 31)	P-Value
**Pump time (min)**	126 ± 29	110 ± 26	0.06
**Clamp time (min)**	68 ± 19	64 ± 15	0.4
**Narcotic total dose (mg)**	16.5 ± 4.5	14.2 ± 4.4	0.06
**Benzodiazepine total dose (mg)**	11.1 ± 2.8	10.2 ± 4.4	0.3
**Packed cell transfusion (unit)**	2.1 ± 0.7	1.8 ± 0.9	0.1
**FFP transfusion (unit)**	1.7 ± 0.4	1.6 ± 0.9	0.7
**Urinary output (mL)**	1234 ± 665	930 ± 465	0.04
**Inotropes (micro/kg/min)**			
Epinephrine dose	0.06 ± 0.03	0.06 ± 0.01	0.9
Dopamine dose	10	10	0.9
Norepinephrine dose	0.06 ± 0.02	0.05 ± 0.01	0.4

Abbreviation: FFP, fresh frozen plasma.

Analysis of postoperative vital signs and parameters showed that the heart rate 24 hours after surgery was significantly higher in the dexmedetomidine group compared to the control group (95 ± 10 vs. 87 ± 10 beats/min, P = 0.005). However, other postoperative characteristics, including length of ICU stay, blood drainage, fluid intake, blood pressure, oxygen saturation, central venous pressure, postoperative ejection fraction (EF), vasopressor requirements, and biochemical parameters, were not significantly different between the two groups (Table 3). Complications recorded included atrial fibrillation, cerebrovascular accident, renal, and respiratory complications. The dose of inotropes represents the average inotrope used in all patients, and the row of "Inotrope need" indicates the proportion of patients who received inotropes in the first 24 hours after the operation. The amount of sodium bicarbonate consumed in the first 24 hours as an indicator of the severity of acidosis was recorded based on the number of 50 cc vials of 4.8% product. The total amount of sedatives used in the first 24 hours was exclusively midazolam, and the morphine equivalent table was used to calculate the total amount of narcotics used, which included morphine, fentanyl, alfentanil, pethidine, and methadone (24).

**Table 3.** The Comparison of Post-Operative Characteristics Between Cases and Controls

Characteristic	Case	Control	P-Value
**Intubation time (h)**	12 ± 5	13 ± 4	0.6
**Length of ICU stay (d)**	4.6 ± 1.22	5.3 ± 2.3	0.1
**Post-op EF (%)**	48 ± 9	47 ± 8	0.8
**Occurrence of Complication **	5	2	0.1
**Variables in first 24 hours**			
Epinephrine dose	0.015 ± 0.03	0.009 ± 0.015	0.3
Norepinephrine dose	0.059 ± 0.13	0.03 ± 0.08	0.3
Dopamine dose	0.67 ± 1.6	0.7 ± 2.0	0.8
Inotrope need	0.64 ± 0.48	0.67 ± 0.47	0.7
sodium bicarbonate used	0.83 ± 1.1	0.93 ± 1.18	0.7
HR	95 ± 10	87 ± 10	0.005
SBP (mmhg)	111 ± 9	114 ± 9	0.2
DBP (mmhg)	64 ± 8	64 ± 5	0.7
SPO2 (%)	97 ± 1	97 ± 1	0.9
CVP (cmh2o)	9 ± 2	10 ± 2	0.8
Total sedative used	4.41 ± 8.8	5.8 ± 7.4	0.4
Total narcotic used	1.5 ± 1.8	1.9 ± 2.1	0.3

Abbreviations: HR, heart rate; SBP, systolic blood pressure; DBP, diastolic blood pressure; SPO2, peripheral oxygen saturation; CVP, central venous pressure.

Evaluation of delirium using the RASS demonstrated significantly lower agitation scores in the dexmedetomidine group at 6 hours (P = 0.02) and 24 hours (P = 0.006) after surgery compared to the control group (Table 4). However, when delirium was assessed more objectively using the CAM-ICU, there were no significant differences in delirium incidence between the two groups at 6 hours and 24 hours postoperatively. Interestingly, the difference in non-positive CAM-ICU results was statistically significant at 24 hours for +1 and -1 RASS scores (Table 5). 

**Table 4.** Comparison of RASS Scores Between Cases and Controls

RASS Agitation Score	Case	Control	P-Value
**6-h agitation sedation**			0.02
+3 (very agitated)	0	0	
+2 (agitated)	0	3	
+1 (restless)	1	5	
0 (alert and calm)	10	15	
-1 (drowsy)	3	4	
-2 (light sedation)	10	2	
-3 (moderate)	3	2	
-4 (deep sedation)	3	0	
**24-h agitation sedation**			0.006
+3 (very agitated)	0	0	
+2 (agitated)	2	4	
+1 (restless)	0	5	
0 (alert and calm)	19	21	
-1 (drowsy)	8	1	
-2 (light sedation)	1	0	
-3 (moderate)	0	0	
-4 (deep sedation)	0	0	

Abbreviation: RASS, Richmond Agitation-Sedation Scale.

**Table 5.** Comparison of Confusion Assessment Method Results Between Cases and Controls ^a, b^

Times and RASS agitation Score	Case		Control		P-Value (+)	P-Value (-)
	CAM+	CAM-	CAM+	CAM-		
**6-h agitation sedation**					1.000	0.863
+1 (restless)	0 (0.0)	1 (9.1)	1 (25.0)	4 (20.4)		
0 (alert and calm)	1 (50.0)	8 (72.7)	2 (50.0)	13 (65.0)		
-1 (drowsy)	1 (50.0)	2 (18.2)	1 (25.0)	3 (15.0)		
Total	2 (100.0)	11 (100.0)	4 (100.0)	20 (100.0)		
**24-h agitation sedation**					0.445	0.007
+1 (restless)	0 (0.0)	0 (0.0)	2 (25.0)	3 (15.8)		
0 (alert and calm)	3 (60.0)	16 (72.7)	5 (62.5)	16 (84.2)		
-1 (drowsy)	2 (40.0)	6 (27.3)	1 (12.5)	0 (0.0)		
Total	5 (100.0)	22 (100.0)	8 (100.0)	19 (100.0)		

Abbreviation: RASS, Richmond Agitation-Sedation Scale; CAM, confusion assessment method.

^a^ Values are expressed as No. (%).

^b^ P < 0.05 was considered statistically significant.

## 5. Discussion

In this randomized, double-blind, placebo-controlled trial of 61 patients undergoing CABG surgery, intraoperative dexmedetomidine infusion started after anesthesia induction did not significantly reduce POD rates compared to placebo when delirium was assessed objectively using the CAM-ICU tool. Although previous studies have reported decreased POD with perioperative dexmedetomidine in cardiac surgery patients, these findings have been inconsistent. A recent meta-analysis by Liu et al. found that dexmedetomidine significantly reduced the incidence of POD after cardiac surgery (25), but a large-scale study yielded negative results, raising serious doubts about the preventive effect of dexmedetomidine (26).

Our study employed rigorous methodology with randomization, blinding, validated delirium assessment tools, and a relatively appropriate sample size. Interestingly, while CAM-ICU assessments showed no difference in delirium rates between the groups, the dexmedetomidine group had significantly lower Richmond Agitation-Sedation Scale (RASS) agitation scores at 6 and 24 hours postoperatively. This suggests that dexmedetomidine might subjectively improve patient calmness and cooperation, but may not objectively reduce delirium when measured using tools like CAM-ICU. The difference in non-positive CAM-ICU results for specific RASS scores is also notable and requires further investigation.

The lack of efficacy for delirium prevention could be partially attributed to the lower-than-intended dose of dexmedetomidine used. We used a maximum infusion rate of 0.5 μg/kg/h compared to 0.7 - 1 μg/kg/h in some previous studies. The lower dose may have been inadequate to demonstrate anti-delirium benefits. Higher doses come with risks of bradycardia and hypotension, which we did not observe. Larger trials are needed to find the optimal dexmedetomidine dose that balances safety and efficacy.

Delirium is considered a common, life-threatening medical complication that contributes to heightened morbidity, mortality, and social expenses. Although the exact etiology of the condition has not been determined, several risk factors such as gamma-aminobutyric acid (GABA) receptor agonists, anticholinergic agents, neurotransmitter imbalance, anesthesia, mild cognitive impairment, and sleep deprivation have been identified (27). Recent studies have also highlighted the role of inflammation in delirium pathogenesis (28). Moreover, there is evidence confirming the association of postoperative hypoxemia with the development of delirium (29). Given the complex nature of the condition, innovative methods are urgently needed to prevent it.

Notably, dexmedetomidine plays a unique role that can be quite different from other sedatives used during mechanical ventilation. Several studies have evaluated this drug and its neuroprotective properties (30). Although the exact mechanism of dexmedetomidine's neuroprotective action is not fully defined, Kim et al. suggest that deactivating the TLR-4/NF-kappa pathway may inhibit inflammation (31). According to Bao and Tang, the neuroprotective effects of dexmedetomidine include the regulation of catecholamine release, inhibition of glutamate release, anti-inflammatory and antiapoptotic effects, antioxidant effects, reduction of anesthetic neurotoxicity, and regulation of synaptic plasticity (32). These properties justify the role of dexmedetomidine in preventing delirium, managing pain, and reducing the dose of associated drugs that increase delirium risk (e.g., narcotics).

A recent meta-analysis demonstrated that applying dexmedetomidine without a loading dose can significantly decrease the incidence rate of delirium (19). Another study revealed that low-dose (0.1 μg/kg/h) dexmedetomidine infusion (without a loading dose) can effectively reduce the incidence rate of postoperative delirium without increasing the incidence of bradycardia and hypotension (33). Moreover, Lin et al. (34) established that high-dose infusion is associated with adverse effects, meaning most adverse events occur during a loading or high-maintenance dose infusion. Therefore, combining the results of these studies, we can conclude that dexmedetomidine infusion without a loading dose can be quite useful in reducing the incidence of cardiovascular adverse effects as well as the incidence of delirium itself.

Unfortunately, due to the lack of more detailed studies and exact data, the optimal dosage of dexmedetomidine for preventing delirium cannot yet be identified, which we hope will be revealed in future research.

The most commonly reported adverse events of dexmedetomidine are hypotension and bradycardia, which are associated with its α2 adrenoreceptor agonist mechanism. It was not surprising that the incidences of hypotension and bradycardia were not higher in the dexmedetomidine group in our trial, as we used the minimum recommended dose of the drug. This could also explain the lack of effect in reducing the incidence of delirium. However, our study did not align with previous studies regarding these complications. Other complications, including renal and respiratory issues, atrial fibrillation, and cerebrovascular accidents, as shown in Table 4, had a limited incidence and no significant difference between the two groups.

It can be carefully interpreted from Table 4 that in the postoperative period and the short term, 24 patients were in an optimal cognitive state, i.e., calm and alert, with more patients in the control group than in the dexmedetomidine group. However, after 24 hours, this number increased to 40, with the two groups being almost equal. Regarding restlessness and agitation, both in the short term and long term, the number of patients in the control group was significantly higher than in the dexmedetomidine group. Conversely, for drowsiness and sedation, particularly in the short term, the number of dexmedetomidine recipients was much higher. In the long term, the dexmedetomidine group was gradually limited to a drowsy state, which is close to the ideal condition.

Our study has strengths and limitations. In most studies, dexmedetomidine has been prescribed after the occurrence of delirium and as a prophylaxis in the ICU. The strength and distinguishing point of the present study was the use of this drug from the time of anesthesia induction to the end of the operation, without a loading dose, to mitigate its side effects. We acknowledge that this research is not reliable in terms of evaluating differences in mortality and other complications. However, considering the randomized design used in this study and the fact that it is one of the first studies where dexmedetomidine infusion occurs at anesthesia induction, we believe that we have enhanced the effect of the substance, resulting in the reported positive findings. The data we collected were on vasopressors and inotropes, with vasodilators playing no role. We evaluated pain at ICU discharge as a routine but did not collect data on the amount or details of painkiller usage.

### 5.1. Conclusions

Our clinical trial on cardiac surgery patients suggests that starting dexmedetomidine before CPB as a supplement to inhalation anesthesia improves patient calmness and cooperation postoperatively. However, it may not objectively reduce the delirium rate when measured using tools like the CAM-ICU. Further studies with larger sample sizes are needed to clarify the efficacy of perioperative dexmedetomidine on postoperative delirium.

## Data Availability

The dataset presented in the study is available on request from the corresponding author during submission or after publication.

## Clinical Trial Registration Code:

IRCT20180724040575N4.
